# Progress in the brain–computer interface: an interview with Bin He

**DOI:** 10.1093/nsr/nwz152

**Published:** 2019-10-12

**Authors:** Chengyu Li, Weijie Zhao

**Affiliations:** 1 Principal Investigator, Institute of Neuroscience, Chinese Academy of Sciences; 2 NSR news editor based in Beijing

## Abstract

*What can the brain–computer interface (BCI) do? Wearing an electroencephalogram (EEG) headcap, you can control the flight of a drone in the laboratory by your thought; with electrodes inserted inside the brain, paralytic patients can drink by controlling a robotic arm with thinking. Both invasive and non-invasive BCI try to connect human brains to machines. In the past several decades, BCI technology has continued to develop, making science fiction into reality and laboratory inventions into indispensable gadgets. In July 2019, Neuralink, a company founded by Elon Musk, proposed a sewing machine-like device that can dig holes in the skull and implant 3072 electrodes onto the cortex, promising more accurate reading of what you are thinking, although many serious scientists consider the claim misleading to the public*.

*Recently, National Science Review (NSR) interviewed Professor Bin He, the department head of Biomedical Engineering at Carnegie Mellon University, and a leading scientist in the non-invasive-BCI field. His team developed new methods for non-invasive BCI to control drones by thoughts. In 2019, Bin’s team demonstrated the control of a robotic arm to follow a continuously randomly moving target on the screen. In this interview, Bin He recounted the history of BCI, as well as the opportunities and challenges of non-invasive BCI*.

## The past and future of BCI

**NSR:** How would you introduce BCI to the general public?

**He:** I think BCI includes two aspects. The first one is what the public are currently interested in: we can detect and decipher the brain signals, and use them to control machines, such as robotic arms, drones or computers. The other aspect is that, instead of passively detecting brain signals, we can modulate the activity of neural networks in the brain by electric, magnetic or acoustic stimulation. Such neuromodulation techniques have already been used for treatment of diseases.

**NSR:** What are the major milestones in the history of BCI?

**He:** The concept of BCI was proposed by Jacques Vidal in the 1970s. After that, one of the major developments is that scientists can use EEG signals to noninvasively control a cursor on the screen by thought. This achievement invoked much interest from the scientific community as well as the public. The governments and funding agencies also became willing to invest in this field. In a 2004 *Proceedings of the National Academy of Sciences of the United States of America* paper, Jonathan Wolpaw and colleagues brought another breakthrough for non-invasive BCI. Instead of the one-dimensional movement in the past, they were able to control the cursor to move two-dimensionally. After that, many non-invasive-BCI laboratories have focused on pushing the non-invasive-BCI technologies controlling virtual or physical objects. My own lab has been focusing on achieving non-invasive-BCI control of more realistic actions of an object, such as a virtual helicopter, a drone and a robotic arm, beyond the computer-cursor movement.

In the field of invasive BCI, Miguel Nicolelis of Duke University was an early worker to implant electrodes into monkey brains and the monkeys were able to control cursors by thoughts. Andrew Schwartz and colleagues at the University of Pittsburgh have demonstrated, on both monkeys and humans, controlling of both virtual cursors and physical robotic arms. Now, researchers in Brown University, Stanford, UC Berkeley, Carnegie Mellon, Caltech and many other institutions are working on invasive BCI and have many new advances.

Currently, there is a new direction for invasive BCI, which is bidirectional BCI. When the robotic arm touches an object, the sensor can feedback this information to the brain by electrically stimulating the somatosensory cortex of the participant, so that the participant can truly feel that he or she is touching the object, but not acknowledged merely by watching the robotic arm.

**Figure 1:**
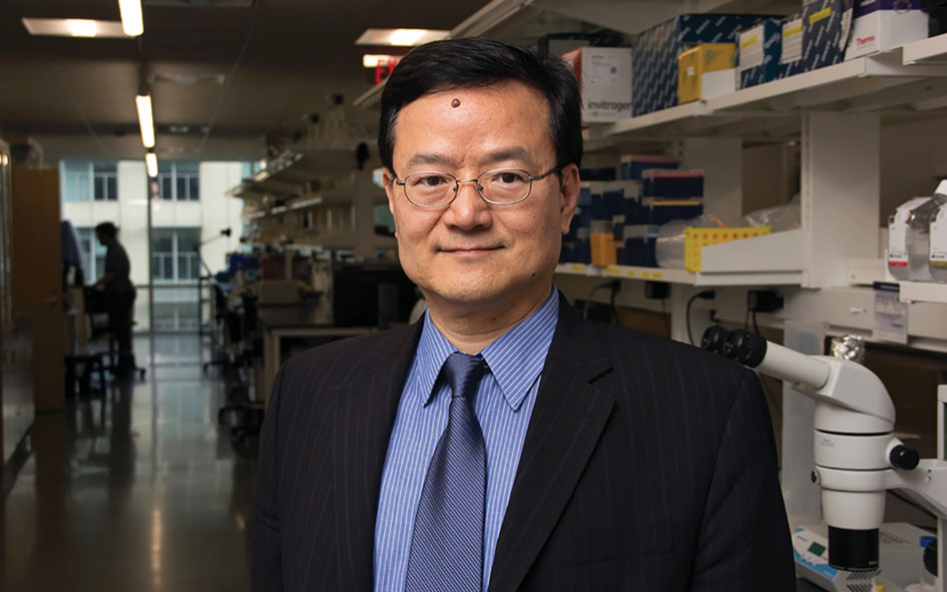
Professor Bin He is the department head of Biomedical Engineering at Carnegie Mellon University, and a leading scientist in the non-invasive-BCI field *(Courtesy of Professor Bin He).*

**NSR:** How did you enter the field of BCI?

**He:** My original research direction is neural imaging. Nearly 20 years ago, I became interested in BCI when I was working in the University of Illinois. At that time, there was much doubt about the feasibility and the future of this young technology. Many researchers did not fully believe it. As a scientist, I thought that instead of anxiously waiting for experimental results from other laboratories, it would be better to test it immediately by myself. So I started to enter this field and soon found it very interesting. Throughout the years, I moved from the University of Illinois to the University of Minnesota, and then to Carnegie Mellon two years ago, with BCI continuously my research focus. Besides being motivated by curiosity, I was also motivated by the expectation that BCI can actually benefit patients and improve the quality of their lives. I think most BCI researchers have the same expectation.

**NSR:** Have you experienced BCI yourself?

**He:** No. I know that many scientists experiment on themselves. But my practice is never to be a participant of my own experiments so that I would not be influenced by subjective bias. I am not sure whether this is right or not, but it is true that I have never been a participant in any experiments.

## Non-invasive BCI: more than a ‘rough black box’

**NSR:** What are the neural signals detected and deciphered by EEG-based non-invasive BCI?

**He:** There are two major kinds of non-invasive BCI. The first one is based on motion imagination. We ask a participant to imagine the motion of his or her own limbs and record the EEG signal. The outcome is that when the participant imagines the motion, the robotic arm or other devices moves with his or her imagination. Wolpaw’s 2004 work on two-dimensional cursor movement and the works in my lab are of this kind. The other kind of non-invasive BCI is based on event-related potential (ERP). Scientists record different types of ERP, such as P300, steady-state visually evoked potential (SSVEP) and auditory evoked potential (AEP), then use these signals to control the machines.

**NSR:** Is the spatial resolution of non-invasive BCI lower than that of invasive BCI?

**He:** That’s right. Non-invasive BCI cannot compare with invasive BCI in the senses of accuracy and directness. There are around 100 billion neurons in a single human brain. It is impossible for EEG to record the activities with single-neuron resolution. But I think we can consider this issue from another angle. Brain’s functional activities are the results of neural networks formed by large numbers of synchronized neurons, and this coordinated functional activation can be recorded by EEG. So you can say that EEG is unable to record activities of single neurons; but you can also say that EEG filters out single-neuron activities and record synchronized network activities of the brain that may be more functionally meaningful. From this point of view, EEG, magnetoencephalogram (MEG) and other non-invasive methods all have their advantages and disadvantages simultaneously. They have unique capability in recording brain function.

Moreover, invasive techniques have difficulties in application. It is not easy to apply invasive BCI even for patients, not to mention for healthy people. Non-invasive BCI has advantages for application and is getting more and more recognized and supported by the National Institute of Health (NIH) and other institutions. There are many BCI researchers throughout the world, focusing on both invasive and non-invasive BCI.

The EEG-BCI community has a very good tradition that many laboratories, including my own, open the recorded EEG-BCI data to the world.

—Bin He

**NSR:** Are there more non-invasive-BCI researchers than invasive-BCI researchers?

**He:** I suppose so, because the entry level of non-invasive BCI is relatively low. To perform experiments, you need only an EEG headcap and a set of EEG device. If you do not perform experiments and only want to optimize the algorithms, it is even easier. The EEG-BCI community has a very good tradition that many laboratories, including my own, open the recorded EEG-BCI data to the world. So any graduate student in any country can download the data and develop new algorithms.

**NSR:** Deep learning can help with EEG data analyses. But the problem is: is the EEG analysis process becoming a black box? We do not need to know which are the brain regions generating the signals or what are the actual meanings of the signals, and the only thing needed is to fit a large amount of data into the deep learning algorithm?

**He:** Deep learning is really helpful for brain data analyses, especially when we are unable to match the signals with specific neurons. I think the next-generation scientists should master machine learning as a basic skill.

But the EEG deciphering process is not completely a black box. We are trying to understand the information and find out the connections between EEG signals and brain activities. Currently, the state-of-the-art source localization and source imaging technologies can localize the signal source with a resolution as high as five millimeters. There are still a huge number of neurons within five millimeters, but this resolution would have been unimaginable 20 years ago and is useful for simple clinical applications. We can also combine neural modulation and neural imaging, because imaging after perturbation can reveal how the brain responds and what are the signals generated under specific stimulation conditions.

**NSR:** Are there any significant developments for EEG-signal detection?

**He:** Scalp electrodes did not evolve greatly in the past years. We use EEG headcaps in laboratory research, which is not very pleasing to the eye. Some companies have developed well-designed head rings. They look prettier and are more acceptable to customers, but the underlying technologies are the same. Comparing with the advances in software and algorithms, the EEG-detection hardware did not change much.

**Figure 2:**
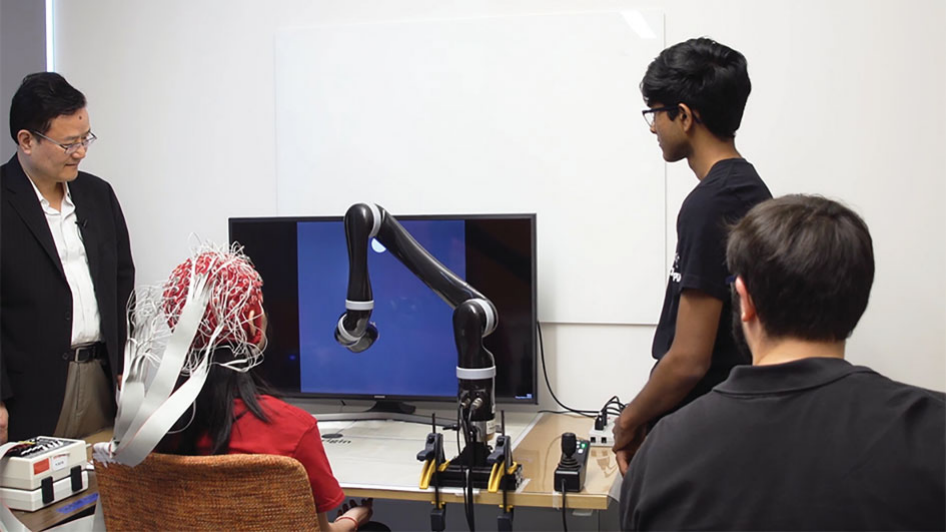
Bin He and his students working in the lab. The participant wearing an EEG headcap is controlling a robotic arm by thought *(Courtesy of Professor Bin He)*.

## Bidirectional BCI: modulating the brain

**NSR:** You mentioned bidirectional BCI, which can feedback tactile information to the brain while guiding the movement, thus forming a closed loop. How is the research status of this direction?

**He:** This is a hot topic for invasive-BCI researchers. Many labs are working on it. Its recording and feedback stimulation are both performed directly on the cortex, so it has the potential to do these things. Scientists working on non-invasive BCI are also attempting to achieve a similar goal, but much still needs to be done to be able to target specific brain regions using a wearable device. Novel non-invasive neural stimulation techniques will be needed to move the research forward.

**NSR:** Broadly speaking, can we consider the brain-stimulation methods used in the clinics as BCI technologies?

**He:** Yes, deep brain stimulation (DBS) can be considered as BCI broadly. I would like to introduce something my lab is working on. The traditional brain-stimulation treatments apply electric or magnetic stimulation on the brain. But there is a limitation here. Electric and magnetic stimulations are non-focal according to the Poisson Equation and the Maxwell’s Equations, because there is the problem of volume conduction. We are trying to stimulate the brain noninvasively using ultrasound signals, which are very focal and can reach deep brain regions. If this could be realized, even though the new method may be not as effective as DBS, it can offer a non-invasive alternative for the patients.

**NSR:** There have already been ultrasound facilities used in hospitals that can generate brain lesions. So what you want to do is not lesion, but modulation?

**He:** Yes, that’s right. A number of laboratories in several countries are also working on this direction of low-intensity focused ultrasound neuromodulation. I personally consider it to be promising.

## BCI and neuroscience

**NSR:** The brain is the common research target of BCI and neuroscience. But neuroscience’s help in non-invasive BCI seems to be limited.

**He:** Yes. Neuroscience connects tighter with invasive BCI. They both perform accurate operations on neurons and share a number of experimental methods. But I think for BCI technology as a whole, its long-term development cannot be guaranteed without the participation of neuroscience, engineering, computer science, materials science and many other fields.

**NSR:** Are non-human primates widely used in BCI research? We may need to experiment on monkeys before we experiment on humans.

**He:** To control machines by thought, invasive-BCI researchers had first performed experiments on monkeys before they began to implant electrodes into human brains. But for non-invasive BCI, most behavior experiments are directly performed on humans because it is not easy to do such experiments on monkeys. It is difficult to put an EEG hat on the monkey’s head. It is also difficult to inform the monkeys the experiment’s goal and let them cooperate with you. I tried to experiment on monkeys when I was in the University of Minnesota, and it was really difficult.

But, of course, if we want to develop BCI technologies that modulate the brain with external signals, it is a natural choice to do animal experiments (including but not limiting to monkeys) before human experiments.

## Prospect: BCI-research directions

**NSR:** What will be the next BCI breakthroughs?

**He:** For invasive BCI, many laboratories are working on bidirectional BCI. As a bystander, I think there will be major breakthroughs on this topic within five to ten years.

For non-invasive BCI, the EEG-signal-analysing methods will be further improved. And I think we should not do merely computation; experimentation is extremely important. You have to push the field forward by advancing experiments, not just advancing computational algorithms.

To create technologies and products that can actually benefit people, we need more young researchers entering this field. BCI is a multidisciplinary field. We need talents with multiple backgrounds including but not limited to neuroscience, engineering, computer science and materials science for both invasive and non-invasive BCI.

**NSR:** How are Chinese scientists doing in BCI research?

**He:** Many Chinese researchers are working on this field and have achieved many good results. I personally know two Chinese BCI groups: the groups in Tsinghua University and Zhejiang University, among others. The Tsinghua group developed the novel SSVEP paradigm for non-invasive BCI. The Zhejiang University group works on invasive BCI and has accomplished a number of interesting works.

## BCI may change patients’ life within 20 years?

**NSR:** There are many innovative companies and investors interested in BCI. Have you formed your own company?

**He:** I myself focus on basic laboratory research due to my deep interest in basic research and many other responsibilities. I dare not say what will happen in the future. But at this moment, I have not formed my own company and am not actively working on BCI commercialization.

**NSR:** What do you think of Musk’s Neuralink technology?

**He:** If the news reports are accurate, I think Neuralink’s work is a very important technological breakthrough, which is a great step forward comparing with the current laboratory works. In the future, this technology is possible to be used on epilepsy patients who need surgical intervention. It is now a common clinical practice to perform invasive recording and stimulation procedures on these patients and Neuralink’s work may provide a better choice comparing with the current technologies. But again, I suppose that invasive technologies are difficult to be applied in common people, regardless how thin an electrode wire could be. Non-invasive BCI has wider application possibilities.

**NSR:** Will there be ethical problems about the application of BCI?

**He:** Yes. In the US, there has been much discussion on the ethical issues of BCI. If we want to do neural manipulation on human brains, privacy concerns and other ethical problems will be raised. On the other hand, all of the invasive technologies face problems such as how to evaluate their harms to the human body and in which conditions it can be considered necessary for surgeries. All of these issues need to be further discussed.

**NSR:** How will BCI change our daily lives within 20 years?

**He:** In the history of BCI, major progress appears every five years. Twenty years later, I think the major applications of BCI will be in the medical field. By that time, brain-controlled artificial limbs, wheelchairs and robotic arms will be able to enter the daily lives of disabled and paralysed patients. With these BCI devices, they will be able to move, eat and control external equipment all by themselves. Their life quality will be greatly improved. Currently, prototypes of these kinds of devices have already appeared in laboratories. But we need some time to make the devices reliable and robust before they can be commercialized. The good news is that many scientists are working on BCI; governments and many investors are also willing to support the BCI industry. So I think it is very likely that these products can be commercialized within 20 years.

Healthy people do not need brain controlled robotic arms to eat their dinner.

—Bin He

On the other hand, we cannot do everything with BCI. Healthy people do not need brain-controlled robotic arms to eat their dinner but may benefit from the capability of brain control of many devices in one's environments. BCI products should focus on the requirements which are difficult to be achieved by other methods but can be achieved by BCI. During the development of the BCI industry, society expectation and BCI products need to meet with each other to achieve a final balance.

## Notes

Chengyu Li is a Principal Investigator at the Institute of Neuroscience, Chinese Academy of Sciences; Weijie Zhao is an NSR news editor based in Beijing.

